# Experimental neutron scattering evidence for proton polaron in hydrated metal oxide proton conductors

**DOI:** 10.1038/ncomms15830

**Published:** 2017-06-14

**Authors:** Artur Braun, Qianli Chen

**Affiliations:** 1 Modern Materials and Surfaces, Laboratory for High Performance Ceramics Empa, Swiss Federal Laboratories for Materials Science and Technology, Uberlandstrasse 129, Dübendorf 8600, Switzerland; 2 Department of Physics, ETH Zürich, Zürich 8057, Switzerland; 3 State Key Laboratory of Metal Matrix Composites, University of Michigan-Shanghai Jiao Tong University Joint Institute, Shanghai Jiao Tong University, Shanghai 200240, China

## Abstract

Hydration of oxygen-deficient metal oxides causes filling of oxygen vacancies and formation of hydroxyl groups with interstitial structural protons, rotating around the oxygen in localized motion. Thermal activation from 500 to 800 K triggers delocalization of the protons by jumping to adjacent oxygen ions, constituting proton conductivity. We report quantitative analyses of proton and lattice dynamics by neutron-scattering data, which reveal the interaction of protons with the crystal lattice and proton–phonon coupling. The motion for the proton trapped in the elastic crystal field yields Eigen frequencies and coupling constants, which satisfy Holstein’s polaron model for electrons and thus constitutes first experimental evidence for a proton polaron at high temperature. Proton jump rates follow a polaron model for cerium-oxygen and hydroxyl stretching modes, which are thus vehicles for proton conductivity. This confirms that the polaron mechanism is not restricted to electrons, but a universal charge carrier transport process.

## Abstract (web summary)

Hydration of oxygen vacancies could form hydroxyl groups with interstitial structural protons. Here a quasi-elastic neutron scattering study reveals proton polarons in proton-conducting ceramic electrolytes, and the proton transport turns out to be a cooperative process.

The proton is a structural element in hydrocarbons, metal hydrides and water. It is an elusive charge carrier^1^ and the energy carrier in the hydrogen economy. As a structural element it is in a localized state; whereas as a mobile ion and as an energy carrier it should have a delocalized nature.

Numerous metal oxides have proton-conducting properties and many rare earth transition metal oxides with perovskite structure are good proton conductors. Some of them are prospective solid electrolytes at temperatures from 500 to 800 K^2^. The temperature-dependent proton conductivity is paralleled by substantial changes of the chemical and molecular structure^3^. Experimental studies show that the proton conduction is an Arrhenius-type thermal activated process, phenomenologically reminiscent of a polaron-type electronic conductivity^4^, which is observed in many 3*d* transition metal oxides with perovskite structure^5^.

Therefore, a proton can be pictured as a trapped charge in an elastic crystal field and thus at least conceptually satisfy the definition of a polaron, which has been proposed and mathematically exercised specifically by theorists Samgin^6^ and in more general terms by Krasnoholovets *et al*.^7^. Observations on the elastic properties made by experimenters Slodczyk *et al*.^8^ with vibration spectroscopy made them speculate over the potential polaronic nature of the proton conductivity: ‘The question of the vibrational signature of isolated proton (for example, the ionic proton, a proton sharing its interaction with more than two acceptors) and its dynamic nature (proton gas, polaron and so on) is open’^8^. Yet, such a proton polaron was not identified so far in hard inorganic matter such as metal oxides.

Here we determine the proton jump times of hydrated yttrium substituted barium cerate with neutron-scattering and electroanalytical methods. The proton jump times follow a temperature dependence that fits the mathematical model of a polaron. Moreover, comparison with our vibration spectroscopy data suggest that it is the cerium-oxygen and hydroxyl stretching modes that propel the protons as charge carriers.

## Results

### Pressure-dependence of proton diffusion

**Figure 1:**
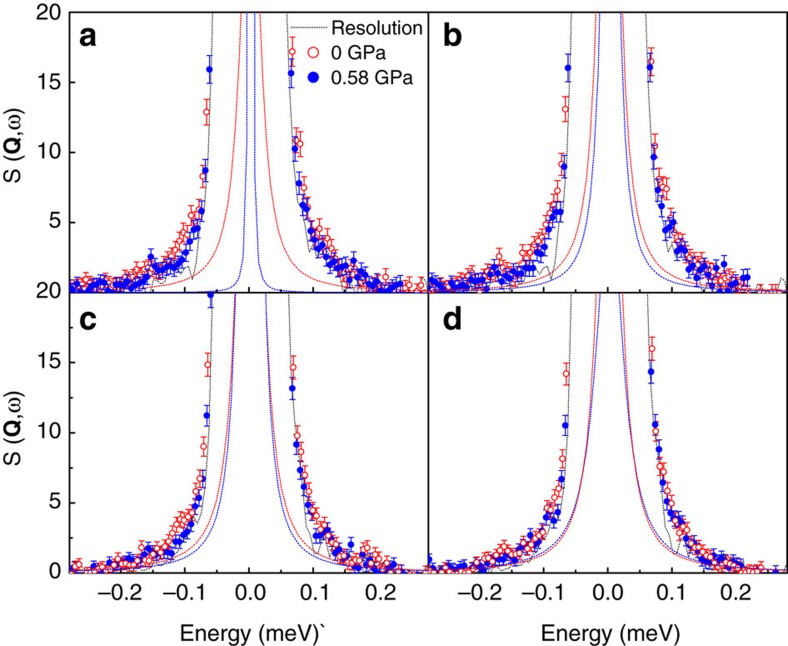
Pressure effect on neutron spectra. QENS curves of hydrated BaCe_0.8_Y_0.2_O_3–*δ*_ (BCY20) obtained in a high-pressure INCONEL cell at 0 GPa (red open data points) and at 0.58 GPa (blue filled data points) pressure at scattering vectors of **Q**=0.6 Å^−1^ at (**a**) 720 K and (**b**) 770 K, and 0.8 Å^−1^ at (**c**) 720 K and (**d**) 770 K. The drawn lines are least square fit Gaussians accounting for the elastic contributions.

We have investigated the proton dynamics in hydrated BaCe_0.8_Y_0.2_O_3−*δ*_ (BCY20) with quasi-elastic neutron scattering (QENS) in a specially designed cell for neutron-scattering experiments at high pressure and high temperature^9^. Figure 1 shows the QENS spectra of pressurized (*P*=0.58 GPa) and non-pressurized (*P*=0 GPa) hydrated BCY20 at 720 and 770 K, respectively, at neutron momentum transfer (scattering vector) **Q**=0.6 Å^−1^ and **Q**=0.8 Å^−1^.

**Figure 2:**
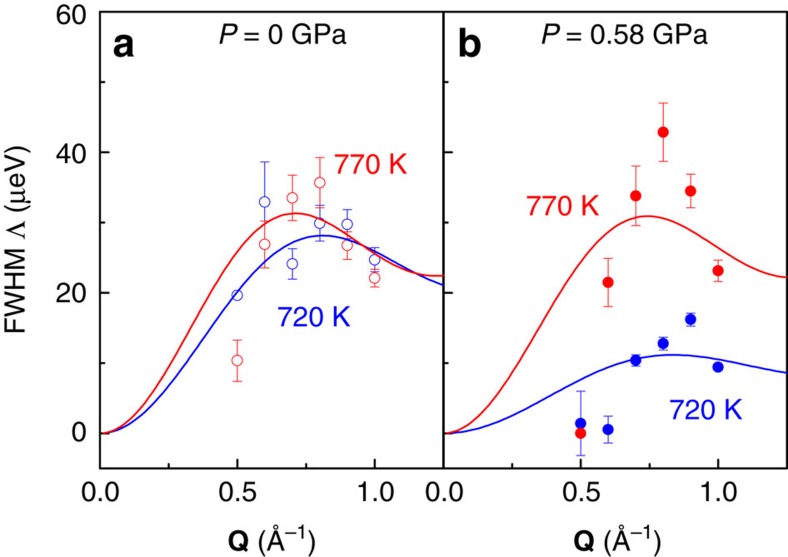
Accordance with Chudley–Elliott proton jump model. (**a**) *P*=0 GPa. (**b**) *P*=0.58 GPa. **Q**-dependence of the Lorentzian full-width-half-maximum Λ(**Q**) fit with the Chudley–Elliot model for hydrated BaCe_0.8_Y_0.2_O_3−*δ*_ in the high-pressure INCONEL cell with pressure (closed data points) and without pressure (open data points), at 720 K (blue data points) and 770 K (red data points). The solid lines are the least square fits of the Chudley–Elliott model (equation (1)) to the data points.

In comparison with the neutron-scattering curves measured from a specimen under no pressure, the QENS line broadening in the high-pressure measurement is clearly noticeable. We can resolve and separate the QENS line broadening from the elastic scattering and the instrument resolution, as exercised in Fig. 2 in a previous work^10^.

It is worthwhile to note that especially at 720 K, the spectra at around ±0.1 meV are broader for the non-pressurized samples in the cell. This indicates that the QENS contribution from the non-pressurized hydrated BCY20 due to proton diffusivity disappears upon pressurizing the sample. This is a direct experimental proof that forced volume decrease causes suppression of the QENS signature of the protons. In other words, when the crystal lattice is squeezed, the protons are slowed down. This is in line with the report by Azad *et al*.^11^ who reported two distinguishable cubic phases in BaZr_0.9_Y_0.1_O_2.95_: one *β*-phase with higher proton conductivity and larger unit cell and one *α*-phase with lower proton conductivity and smaller unit cell. At 770 K, the QENS difference between the spectra with and without pressure is smaller, because at sufficiently high temperature, 770 K rather than 720 K, the protons resist the pressure and maintain higher diffusivity.

For the separation of the QENS contribution from the elastic neutron-scattering curve, the structure factor *S*(**Q**,ω) is subject to least-square fitting with the convolution of a Lorentzian quasielastic scattering function and the instrument resolution, as exercised in ref. 10. The Lorentzian full-width-half-maximum Λ(**Q**) is presented versus **Q** in Fig. 2 and constitutes a measure for the proton diffusivity. Smaller quasielastic line widths are distinguishable at 720 K for hydrated BCY20 measured at high pressure, which are around a factor of two smaller than the FHWM measured without pressure.

Quantitative analysis of the width Λ(**Q**) from QENS spectra with the jump diffusion model by Chudley and Elliott^12^


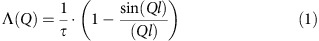


provides numerical results for the proton jump length *l* and time *τ* between two jumps, which yields the proton diffusivity *D* via the relation *l*^*2*^*=*6*Dτ*.

### Activation energy for proton conduction

**Figure 3:**
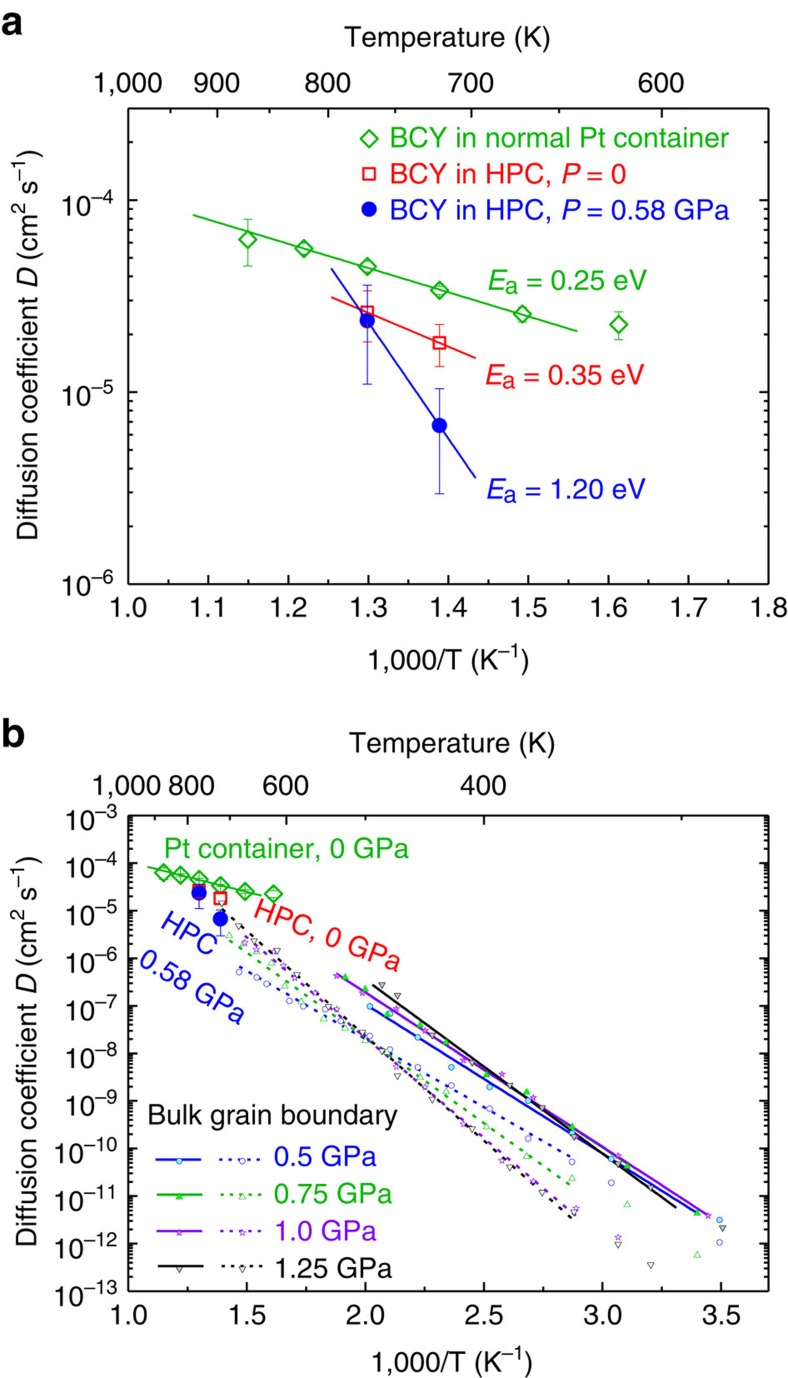
Diffusion coefficients. (**a**) Diffusion coefficients *D*_QENS_ of hydrated BCY20 obtained by QENS at 0 GPa (open red squares) and 0.58 GPa (filled blue dots) in the INCONEL high-pressure cell, and at 0 GPa (open green diamonds) in a Pt container in the temperature range from 620 to 870 K. Data were obtained by least-square fits of Λ(**Q**) to the Chudley–Elliott model. (**b**) Comparison of diffusion coefficients obtained from QENS at 0 and 0.58 GPa (large symbols) and from EIS at 0.5–1.25 GPa (small symbols).

Figure 3 (top) shows the thus obtained diffusion coefficients *D* in Arrhenius representation vs reciprocal temperature. The slope drawn from the two diffusion coefficients obtained from the QENS of hydrated BCY20 powder in the INCONEL cell at no pressure yields an activation energy *E*_a_ of 0.35 eV (ref. 9). The corresponding data from the same powder under 0.58 GPa pressure yield a substantially higher value of *E*_a_=1.20 eV. For comparison, the diffusivity coefficients from an independent QENS measurement on hydrated BCY20 sintered slabs were measured in a Pt container—the standard containment for QENS studies. The sintered slabs have obviously the lowest activation energy and an overall higher diffusion coefficient. As the diffusivity *D* can be expressed by the conductivity *σ* via the Nernst–Einstein relation, we can transform the conductivities from electrochemical impedance spectroscopy (EIS) and compare them with the QENS data:


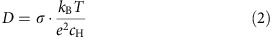


with Boltzmann constant *k*_B_, temperature *T*, elementary charge *e* and concentration of protons *c*_H_ per unit volume. In Fig. 3 (bottom), the diffusivities extracted from high-pressure EIS data from ref. 13 are presented for comparison with the QENS data.

The change of the slope upon pressurizing the specimen in Fig. 3 (bottom) confirms also from the EIS data that the proton diffusion activation energy *E*_a_ increases with pressure. With increasing pressure on the specimen, the protons must overcome a higher activation barrier. This, together with our experimental findings for the proton conductivity at high pressure^9^,^13^,^14^, proves that the activation energy depends on the lattice strain parameter, which in turn is modulated by the elastic properties^15^.

Higher activation energy in a smaller crystal lattice suggests that one single classical hopping process such as the Grotthuss mechanism^2^ alone cannot explain our observation. Rather, cooperative processes such as lattice vibrations, that is, phonons, would assist in the proton transport.

The mobility of protons has been rationalized as a consequence of phonon-assisted jumps^16^,^17^. Proton conductivity has been interpreted as a polaron-activated process^7^. Tomoyose *et al*.^18^ found that the wave function of a proton between two adjacent oxygen ions in an ABO_3_-type perovskite would spread over the double well potential if the O–O separation was lower than 2.9 Å, with the result that the proton would transfer between the energy minimum sites^18^. They derived the activation energy for this process from a small polaron model.

In view of such striking analogy with the polaron-driven electric conductivity in some metal oxides, which manifests in the exponential *T* dependency of *σ* (so-called Holstein mobility contribution^19^), we considered a polaron-type charge transport also for our proton conductor. We follow in so far the spirit of Holstein^4^,^20^,^21^, and Yamashita and Kurosawa^22^ whose work on polaron theory was inspired by the same observation^19^.

The Debye temperature for BaCeY-oxide is *θ*=400 K (ref. 23). Holstein^4^ shows that for *T* >1/2*θ* the motion of the charge carrier is predominantly a diffusion process based on random jumps between neighbouring sites associated with Holstein’s off-diagonal transition. We are therefore considering the high-temperature region of Holstein’s small polaron theory.

### Proton polaron

**Figure 4:**
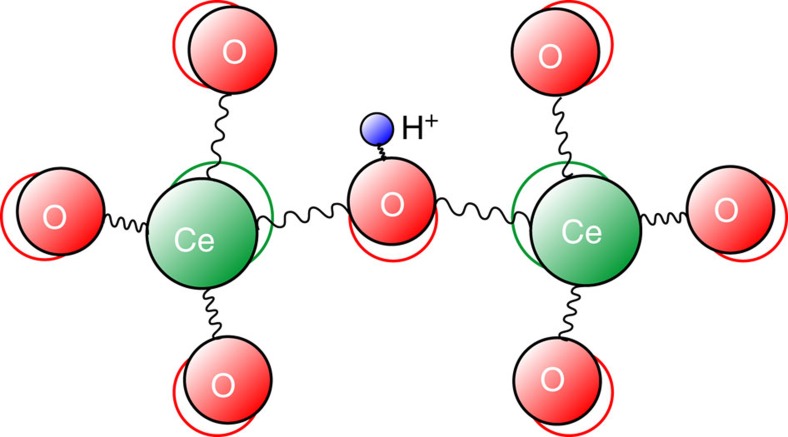
Direction of interaction in crystal lattice. Sketch of a proton (blue sphere) in the lattice of Ce–O in the small polaron model.

The polaron concept was originally introduced by Landau for the description of charge transport in crystal lattices^24^, in particular for electrons, and developed further by other researchers^7^,^19^,^25^. Polaron theory was applied later to distinct different problems such as hydrogen diffusion in metal^26^, hydrogen-bonded systems such as ice^27^,^28^ and perovskites^29^,^30^,^31^, for example. The proton with small ionic radius may have strong bonds with the lattice vibrations. When a proton is introduced into an ionic crystal and moves through the crystal lattice, because of their electrostatic interactions, the lattice ions respond to the proton motion by local lattice relaxation (polarization)^32^ as sketched in Fig. 4. The term ‘polaron’ implies that complete assessment of the problem would therefore include the effect of the ionic character on the pressure dependence of vibration frequency.

Protons have an attractive interaction with negative charged O^2−^ and a repulsive interaction with the positive charged Ce^4+^/Y^3+^ in our BCY20 material. Rather than hopping within a static lattice, proton hopping can be understood as modulated by the vibration of the CeO_6_ octahedra or by the O–H vibrations. Hence, the mobility of protons is a result of phonon-assisted jumps^6^.

### Phonon-modulated activation energy

With respect to the model depicted in Fig. 4, it is instructive to tentatively follow a classical approach by Wakamura^33^,^34^, who considers the vibration of a proton and an oxygen ion in the metal oxide host lattice as a damped harmonic oscillator. Accordingly, the relative displacement Δ*u* between a proton with mass *m*_H_ and an oxygen ion with mass *m*_O_ in an electric field with strength *E*_0_ is


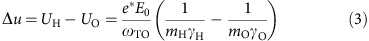


Because of the inverse relation Δ*u* ∼ 1/*ω*_TO_, higher optical phonon frequency *ω*_TO_ will mandate a smaller relative displacement. Assuming that proton hopping with activation energy *E*_a_ is assisted by a large vibration displacement of the oscillator, the activation energy scales parabolic with the optical phonon frequency:


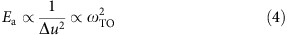


Therefore, we expect pressure dependence for the transversal optical (TO) and also longitudinal optical (LO) modes, and the different contribution of the vibration displacements for the high- and low-energy modes on the polaron transport. The former gives different pressure dependence on the LO modes and TO modes as we would expect from the magnitudes of the Grüneisen parameters for the modes, and the latter gives different influence on the mobile proton.

A change of the crystal unit cell volume will affect the phonon frequencies, the relationship of which is given by the Grüneisen parameter *γ*. The electronic polarization, which is in principle the optical dielectric tensor, affects the frequencies *ω*_TO_ and *ω*_LO_ of the TO modes and LO modes, respectively. Correspondingly, this yields different magnitudes on the Grüneisen parameters for the LO and TO modes, *γ*_LO_ and *γ*_TO_. Wakamura^35^ has demonstrated how different pressure dependencies arise for *ω*_TO_ and *ω*_LO_. In an ionic crystal, *γ*_LO_ assumes a lower value than *γ*_TO_. As the polaron works stronger by the LO mode than by the TO mode, the LO mode must contribute dominantly to the activation energy *E*_a_. It may clarify the difference of parabolic and linear relations of vibration frequency on *E*_a_. Strong contribution of electronic polarizability on *E*_a_ is obvious from the relationship between optical dielectric constant and *E*_a_ values as seen in references^33^,^36^.

Our EIS studies at high temperature and high pressure^13^,^14^ confirm the influence of pressure on the activation energy *E*_a_. At the molecular level, the relation between activation energy and mechanical pressure can be rationalized as the consequence of the shift in the prominent Raman mode *v*_3_, which represents the Ce–O stretching^15^. In other words, the frequency of certain phonon modes can affect the amplitude of the proton motion. At pressure, the spring constant (here not being constant anymore) of the Ce–O stretching bonds increases, which can result in smaller vibration amplitude of the protons. Pressure may keep the protons in the CeO_6_ octahedral cage and raise therefore the energy barrier, that is, *E*_a_ for the proton transfer^9^,^37^.

O–Ce–O bending modes were also observed in the Raman spectra of BCY at lower frequencies. The variation in frequencies for these modes upon pressure, however, was not as significant as the Ce–O stretching modes. In contrast, the pressure-dependent variation for these bending modes was suppressed upon hydration^37^. Therefore, although the O–Ce–O bending modes may contribute to proton transport via the vibration displacement^18^, the effect of these modes is not considered due to its insignificance here.

Supplementary Fig. 1 (top panel) shows the proton conductivity activation energies for pressures up to *P*=2 GPa, which we obtained with EIS from BZY10 (BaZr_0.9_Y_0.1_O_3_) and BCY20, and with QENS from BCY20. For each sample set, the activation energies vary quite linearly with pressure. Based on the linearity of the Raman shifts versus pressure^37^, we can express the pressure by wavenumbers. The bottom panel in Supplementary Fig. 1 shows how *E*_a_ varies linearly with the Raman frequency (shift) from roughly 0.4 eV to around 0.8 eV in the measured pressure range. The flat grey line in Supplementary Fig. 1 (top) is the activation energy derived from equation (13) respectively from equation (14) in ref. 38, after we have expressed the square of the distance, *u*^*2*^, by pressure *p*:


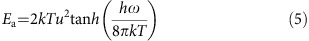


Thus, Samgin’s model predicts a linear relation between *E*_a_ and *p*, which is in line with our experimental data in Supplementary Fig. 1 (top). We find no parabolic relationship in the investigated range of pressure and Raman shift. However, a virtually linear relationship of *ν* and *E*_a_ as was found by Wakamura^33^,^34^,^35^ for a wide range of compounds for the OH stretching frequency^18^ and was found in our study for the Ce–O stretching mode (Supplementary Fig. 1a).

We also find no parabolic relationship between *E*_a_ and *ω*_TO_. Instead, our investigation of the Raman shift under high pressure on hydrated BCY20 (ref. 37) produces a linear relationship between activation energy and phonon mode in line with the derivation by (refs 4, 39)


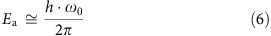


For the elucidation of the difference of parabolic and linear relations of the vibration frequency on *E*_a_, the effects of the ionic character or electronic polarizability would need to be considered. Further work needs to be done to study the difference between the pressure dependence of TO mode and LO mode for a solid conclusion.

### Proton jump time

Holstein^4^ pictured the action of a small polaron as a random walk of a charged particle from site to site. The electron diffusivity is the product of the square of lattice distance and the total non-diagonal transition probability^4^. In analogy to Holstein’s model, Samgin^30^ derived from the hopping mobility of his proposed proton polaron that under the thermal condition *ħω∼kT*, the time *τ* between two subsequent jumps^40^ is


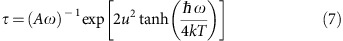


**Figure 5:**
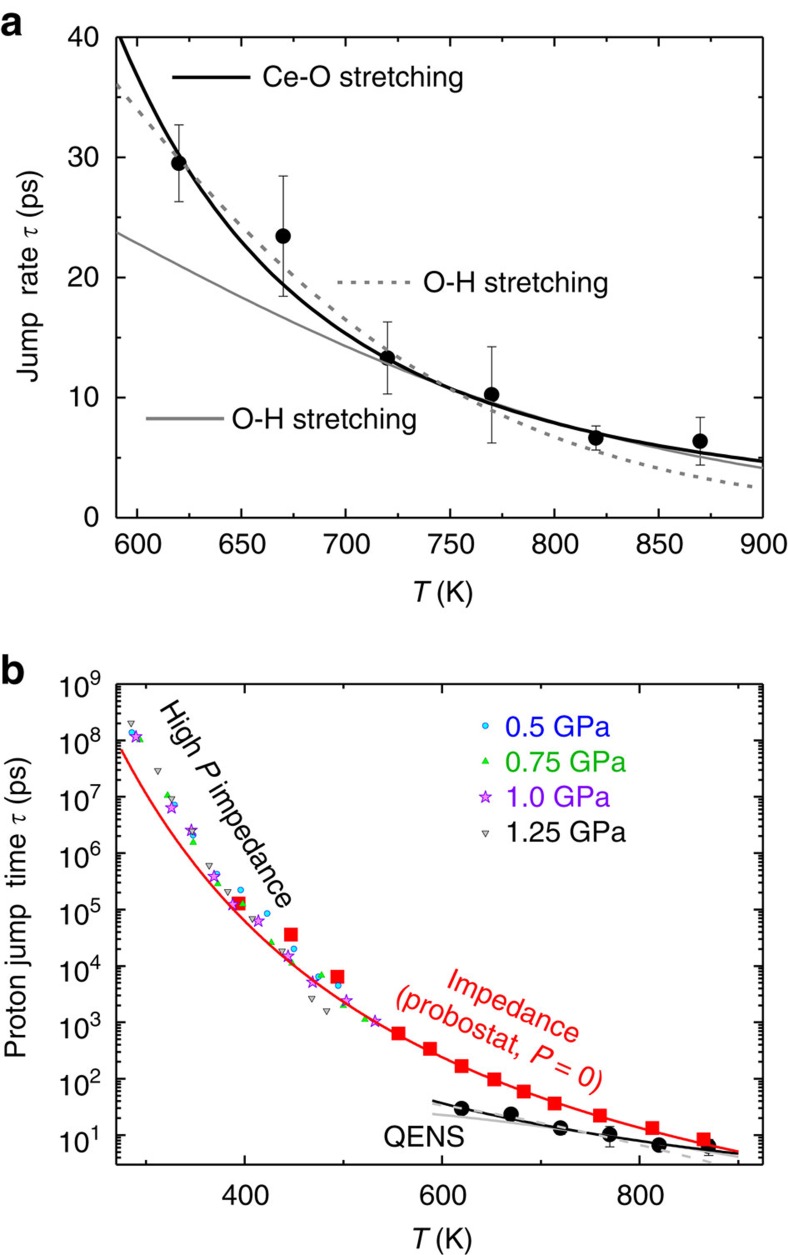
Polaron modelling of proton jump times. (**a**) Time *τ* between two proton jumps obtained from QENS on hydrated BCY20, together with three least square fits of the data points to equation (7). Solid black line represents the Ce–O stretching mode of 355 cm^−1^. Grey dotted and solid lines are O–H stretching mode of 3,560 cm^−1^ (ref. 15) (**b**) Proton jump times obtained from EIS at various pressures using equation (7). Solid red line is the fit using equation (4) and the Ce–O stretching mode (355 cm^−1^) for the filled red squares measured at ambient pressure. The solid red line is the fit using equation (7) and the Ce–O stretching mode (355 cm^−1^) for the proton jump time derived from EIS data at ambient pressure. The fit works well for *T*>500 K and deviates from low temperature data. No proper fitting to the EIS data can be obtained using the O–H stretching mode (3,650 cm^−1^).

*u* is a coupling constant between proton and lattice vibration. With the experimental proton jump time *τ* that we have directly determined by the least square fits from QENS data to the Chudley–Elliott model in ref. 9, we can verify the validity of the mathematical model for Samgin’s proposed proton polaron in ref. 30. Figure 5a shows how the time *τ* between two proton jumps decreases systemically from 30 ps at 620 K to around 7 ps at around 870 K. The drawn lines are least-square fits to equation (7). This is experimental evidence that Samgin’s treatment of the Holstein polaron theory for the proton polaron is applicable to the proton transport studied here, that is, an experimental verification of Samgin’s proton polaron hypothesis and a validation and extension of the Holstein polaron theory.

The proton jump time *τ* can also be extracted from the diffusion coefficients obtained from EIS at various pressures. In the proton jump diffusion model, the following relation holds: *l*^*2*^*=6Dτ*, where *l* is the proton jump length. We estimated the proton jump length from the O–O distance, obtained from high-pressure X-ray diffraction in reference^15^. We extracted *τ* and plot in analogy to the proton jump time determined by QENS, as shown in Fig. 5b. The solid red line is the least square fit of equation (7) using the Ce–O stretching mode (355 cm^−1^) for the proton jump time derived from EIS data at no pressure. The fit, that is, the model reproduces the experimental data points well for *T*>500 K, but deviates from low temperature data. No proper fit of the EIS data can be obtained using the O–H stretching mode (3,650 cm^−1^).

Table 1 summarizes the optimized fit parameters and the obtained ‘proton–phonon coupling’ constant *u* (strictly speaking the coupling constant for the interaction of the proton with the lattice) for the respective vibrational mode. We first discuss a crystal phonon, the Ce–O stretching mode at 355 cm^−1^, shown in the solid green line. The Ce–O stretching mode is the phonon mode, which shows the most significant change in frequency when pressure is applied^15^, that is, the response of this mode to the surrounding environment change is the strongest—the force constant of the Ce–O stretching vibration becomes ‘stiffer’ in a compact lattice. Therefore, when a proton–polaron is brought into the lattice, its interaction of the Ce–O stretching mode could also be strong. In fact, the wavenumber shift of 2 cm^−1^ in this mode^15^ is also the evidence that the phonon vibration changes due to the incorporation of protons. Samgin’s model suggests that the Ce–O stretching mode plays a significant role in the interaction with protons.

The Raman feature at 3,560 cm^−1^ is a local vibrating mode and the signature of the O–H stretching. This mode appears in Raman spectra when the proton is incorporated in BCY20 (ref. 15). The O–H stretching mode obviously promotes proton hopping for *T*>700 K, as illustrated in Fig. 5a. Below this temperature, the solid grey fit line deviates from our experimental jump times. A further fit for *T*<700 K is shown as a dashed grey line in Fig. 5a, resulting in different values for *u* and for a large *E*_a_. This crossover in temperature coincides with other properties for the proton conducting perovskites, such as the discontinuity in the thermal expansion coefficient (second-order phase transition) and the change of the slope for the increase in conductivity versus the temperature^10^.

This discontinuity in thermal expansion and proton diffusion has been observed for the same or for similar materials^41^,^42^,^43^,^44^. Literally, the thermal expansion coefficient decreases in the temperature range where we observe the onset of considerable proton diffusivity and conductivity, that is, here in the range 650–700 K. Most noteworthily, the jump time *τ* is correctly predicted for the Ce–O stretching mode by the proton polaron model (equation (7)), whereas the O–H stretching is validated only for *T*>700 K.

At low temperatures, the proton conductivity will follow a process, which is of quantum-mechanical nature^4^,^20^. Spahr *et al*.^40^ reported that at low temperatures of 10–250 K, proton transport in rutile TiO_2_ is promoted by a transverse oscillation mode, which is coupled by O–H and O–D stretching modes. In the case of the perovskite BaCeY-oxides, protons exhibit a localized rotational motion at low temperatures below 500 K (ref. 45).

Tomoyose *et al*.^18^ suggest a vital role of the bending mode of O–Ce–O in their polaron model. The difference from the Ce–O stretching modes as employed in this work is caused by the different proton diffusion path. Although the O–Ce–O bending modes can reduce the O–O distance within the same CeO_6_ octahedron, the Ce–O B_1g_ and B_3g_ stretching modes as discussed in this work cause a ‘breathing’ motion octahedron^46^, which can lead to shorter distance between next-nearest neighbouring oxygen sites such as O3–O3, as shown in Supplementary Fig. 2. The proton jump length obtained from QENS in ref. 9 suggests the existence of ‘inter-octahedra transfer’ in BaCeY-oxides, which is in line with an investigation using combined first principles and kinetic Monte Carlo simulations^47^. Furthermore, the proton transfer between O3–O3 sites happens only within the *a*–*c* crystal plane, which is in line with our finding that the phonon hardening occurs within the *a*–*c* plane^15^.

**Figure 6:**
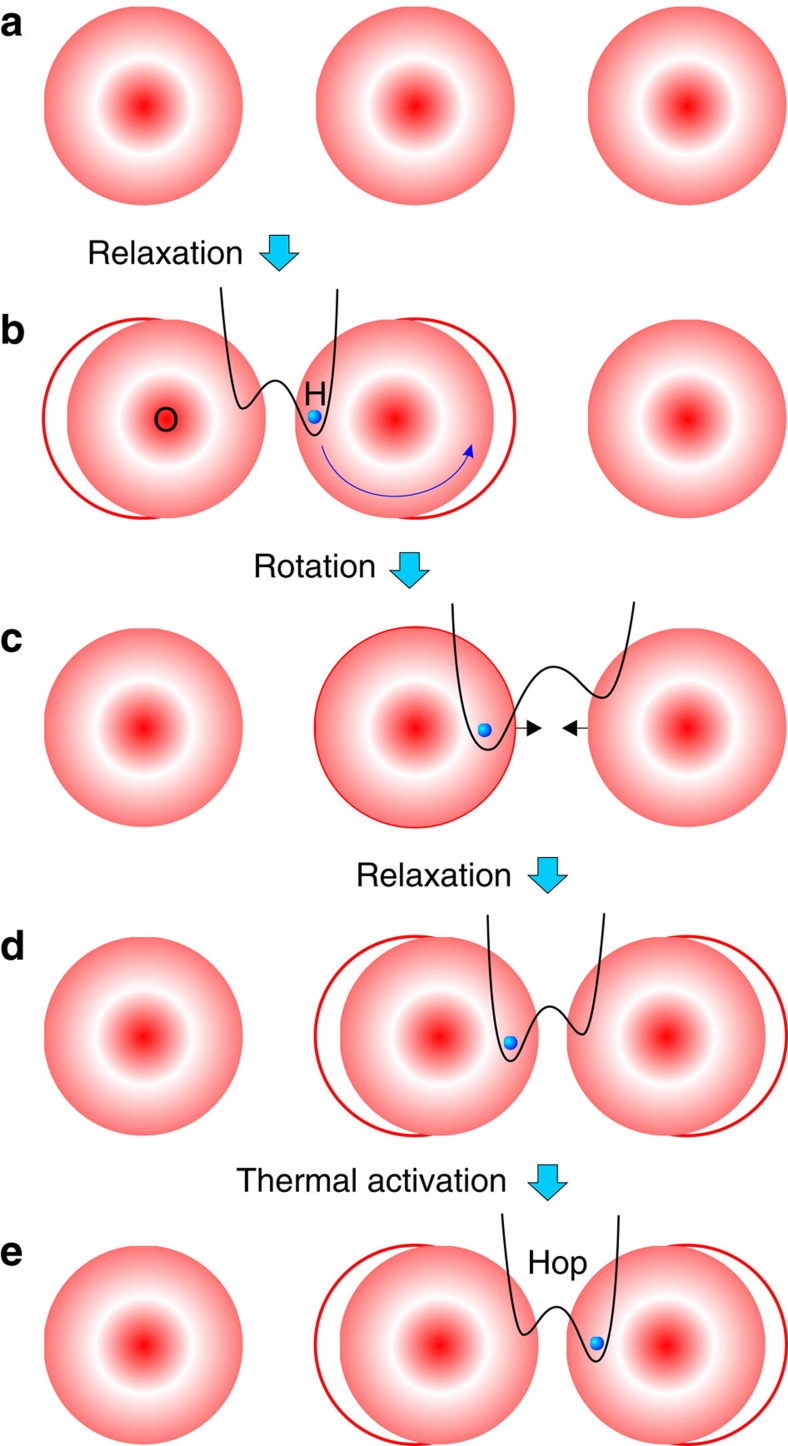
Stages of proton diffusion. Schematic representation of the phonon-assisted hydrogen diffusion process in a long range chain of oxygen. (**a**) Oxygen lattice with no protons. (**b**) Local relaxation of oxygen ions when a proton-polaron is incorporated. (**c**) Proton rotates around the oxygen to a new position. (**d**) The new proton position adjusts the oxygen lattice relaxation. (**e**) Proton hops to the next oxygen site.

Considering the motion of protons in the context of lattice dynamics, proton diffusion is caused by the cooperative atomic displacements. The phonons move mobile species towards the energy barrier. The polaron must overcome the potential barriers, which are posed by the lattice and modulated by its phonons. The protons are considered as hopping at sufficiently high temperatures from site to site due to exchange of energy and momentum with phonons^48^. The steps of phonon-assisted hydrogen diffusion in oxides are detailed in Fig. 6. The local relaxation of the oxygen ions induced by the proton is shown in steps (a) to (b). For long-range transport, a rotation step of the proton around the oxygen is necessary, that is, from (b) to (c). The new position of the proton in (c) results in another relaxation of the oxygen lattice, as shown in (d). The proton overcomes the energy barrier with thermal energy and hops to another oxygen site in (e).

### Effective mass of proton–polaron

Samgin^6^,^30^ considers the proton polaron in a ceramic proton conductor a small polaron, because the range of interaction is smaller than the crystal unit cell, that is, a Holstein polaron. The polaron coupling constant *u* for the relevant Ce–O stretching mode is obtained from the least square fit of Samgin’s model to the experimental data for the proton jump times *τ* from our QENS data and yields *u*=3.87±0.11.

For intermediate polaron coupling with coupling constants *u≈<*4.5, the following relation for the ratio of the polaron mass m* to the band mass *m* of the charge carrier is considered a good approximation^49^:


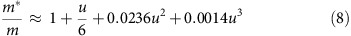


The thus obtained ratio between proton polaron mass and proton mass for the intermediate coupling is m*/m=2.0796±0.0449. Within the weak coupling limit approximation, which includes not the cubic term *u*^*3*^, the ratio is m*/m=1.9985±0.0382. The polaron mass is thus virtually the double proton mass. This outcome may point to the question whether the protons could actually move in pairs and thus constitute a proton bipolaron. A proton may attract the surrounding negative oxygen ions, moving the neighbouring oxygen ions towards the proton. The negative charge density in the vicinity of the proton is therefore increased. This negative charge could attract another proton, causing two protons to pair up. Interestingly, such bipolaron suggestion is consistent with our observation of inter-octahedra transfer in ref. 15 when the protons move in pairs, the nearest oxygen is already occupied by another proton; therefore, the mobile proton proceeds to the next neighbouring oxygen.

## Discussion

In summary, our experimental observation that the proton jump times *τ* are accurately described by Samgin’s proton polaron model lends credibility to the suggestion^6^,^7^,^8^ that the proton–phonon coupling in an ABO_3_ perovskite structure such as BCY20 can be considered the origin for a genuine polaron. In this case, the thermally activated Ce–O stretching mode is the dominant driving force for the coupling of the low-energy phonon mode with the proton diffusion, and thus the physical origin of the proton conductivity in ceramic proton conductors. The coupling strength of this system is within the range of intermediate coupling and yields a polaron mass of virtually exactly m*=*2*. Although this result looks fortuitous, the suggestion that this could be an indication for a process, which is based on protons that move in pairs rather than individually, deserves attention and should warrant further experimental and theoretical investigation.

## Methods

### Sample preparation

The BaCe_0.8_Y_0.2_O_3−*δ*_ (BCY20) powder was prepared by solid-state reaction. Stoichiometric amounts of BaCO_3_ (Sigma-Aldrich, 99%), CeO_2_ (Aldrich, 99.9%) and Y_2_O_3_ (Stanford Materials, 99.9%) were ball-milled in acetone with ZrO_2_ balls of 10 mm diameter in a planetary mill (200 r.p.m.) for 1 h and calcined at 1,000 °C for 12 h twice in succession. The resulting powder was ball-milled again in a planetary mill and then axially pressed at 50 MPa for 0.5 min into monoliths and sintered at 1,400 °C for 24 h. The obtained BCY20 has an average grain size of about 800 nm. For protonation, sintered monoliths or powder was heated to 500 °C in humid N_2_ flow for 24 h, resulting in proton concentration equivalent to [OH]^−^=3.8 mol%.

### QENS at high pressure

QENS on hydrated BCY20 powder were conducted at 720 and 770 K, at the pressure of 0 and 0.58 GPa in a high pressure cell, respectively^9^. QENS experiments were conducted at the neutron wavelength *λ*=5.75 Å at the time-of-flight spectrometer FOCUS, SINQ Neutron Spallation Source^50^,^51^. The detailed cell pressurization, pressure determination procedure were described in ref. 9. QENS data reduction was made with the software package DAVE^52^.

### High-pressure impedance spectroscopy

*In situ* high-pressure EIS were measured on sintered powder at pressures ranging from 0.5–1.25 GPa in a piston-cylinder apparatus^53^ from ambient temperature up to 440 °C. The EIS spectra were simulated in Software Novocontrol WinFit by an equivalent circuit model composed of three parallel RC series of the bulk, grain boundary and electrode responses^13^.

### Data availability

The data that support the findings of this study are available from the corresponding authors upon request.

## Additional information

**How to cite this article:** Braun, A. & Chen, Q. Experimental neutron scattering evidence for proton polaron in hydrated metal oxide proton conductors. *Nat. Commun.* **8,** 15830 doi: 10.1038/ncomms15830 (2017).

**Publisher’s note:** Springer Nature remains neutral with regard to jurisdictional claims in published maps and institutional affiliations.

## Supplementary Material

Supplementary Information

## Figures and Tables

**Table 1:** Proton–phonon coupling constant *u* obtained from least square fit to the lattice-assisted proton transfer model after Samgin, respectively, for the Ce–O stretching and O-H stretching mode^15^.

**Method**	**Assist phonon wavenumber (cm**^**−1**^**)**	**Factor A (cm** **ps**^**−1**^**)**	**Proton-phonon coupling constant** ***u***	**Activation energy** ***E***_**a**_ **(eV)**
EIS	355 (Ce–O stretching)	1.49 × 10^12^	5.29	0.64±0.03
QENS	355 (Ce–O stretching)	0.041	3.87	0.326±0.001
QENS	3,560 (O–H stretching)	7.6 × 10^3^	3.23 (>700 K)	1.29±0.21
QENS	3,560 (O–H stretching)	2.83 × 10^8^	4.00 (<700 K)	1.98±0.35

EIS, electrochemical impedance spectroscopy; QENS, quasi-elastic neutron scattering.

The activation energy *E*_a_ is calculated according to ref. 30.

## References

[b1] Fillaux F. New proton dynamics in solids revealed by vibrational spectroscopy with neutrons. J. Mol. Struct. 511-512, 35–47 (1999).

[b2] Norby T., Wideroe M., Glockner R. & Larring Y. Hydrogen in oxides. Dalton Trans. 3012–3018 (2004). 15452624 10.1039/b403011g

[b3] Chen Q. L. . Observation of oxygen vacancy filling under water vapor in ceramic proton conductors *in situ* with ambient pressure XPS. Chem. Mater. 25, 4690–4696 (2013).

[b4] Holstein T. Studies of polaron motion: Part II. The ‘small’ polaron. Ann. Phys. 8, 343–389 (1959).

[b5] Austin I. G. & Mott N. F. Polarons in crystalline and non-crystalline materials. Adv. Phys. 50, 757–812 (2001).

[b6] Samgin A. L. Lattice-assisted proton motion in perovskite oxides. Solid State Ionics 136, 291–295 (2000).

[b7] Krasnoholovets V. V., Tomchuk P. A. & Lukyanets S. P. Proton transfer and coherent phenomena in molecular structures with hydrogen bonds. Adv. Chem. Phys. 125, 351–548 (2003).

[b8] Slodczyk A., Colomban P., Willemin S., Lacroix O. & Sala B. Indirect Raman identification of the proton insertion in the high-temperature [Ba/Sr][Zr/Ti]O_3_-modified perovskite protonic conductors. J. Raman Spectrosc. 40, 513–521 (2009).

[b9] Chen Q. L. . High-temperature high pressure cell for neutron-scattering studies. High Pressure Res. 32, 471–481 (2012).

[b10] Braun A. . Proton diffusivity in the BaZr_0.9_Y_0.1_O_3−δ_ proton conductor. J. Appl. Electrochem. 39, 471–475 (2009).

[b11] Azad A. K. . Structural origins of the differing grain conductivity values in BaZr_0.9_Y_0.1_O_2.95_ and indication of novel approach to counter defect association. J. Mater. Chem. 18, 3414 (2008).

[b12] Chudley C. T. & Elliott R. J. Neutron scattering from a liquid on a jump diffusion model. Proc. Phys. Soc. Lond. A 77, 353–361 (1961).

[b13] Chen Q. L., Braun A., Yoon S., Bagdassarov N. & Graule T. Effect of lattice volume and compressive strain on the conductivity of BaCeY-oxide ceramic proton conductors. J. Eur. Ceram. Soc. 31, 2657–2661 (2011).

[b14] Chen Q. L. . Hydrostatic pressure decreases the proton mobility in the hydrated BaZr_0.9_Y_0.1_O_3_ proton conductor. Appl. Phys. Lett. 97, 041902 (2010).

[b15] Chen Q. . Effect of compressive strain on the Raman modes of the dry and hydrated BaCe0.8Y0.2O_3_ proton conductor. J. Phys. Chem. C 115, 24021–24027 (2011).

[b16] Hempelmann R. Diffusion of hydrogen in metals. J. Less Common Metals 101, 69–96 (1984).

[b17] Kreuer K. D. Proton conductivity: materials and applications. Chem. Mater. 8, 610–641 (1996).

[b18] Tomoyose T., Shimoji N. & Wakamura K. Proton diffusion in perovskite-type oxides based on small polaron model. J. Phys. Soc. Jpn 74, 3011–3015 (2005).

[b19] Lang I. G. & Firsov Y. A. Calculation of activation probability for a jump of a small-radius polaron. Sov. Phys. JETP USSR 27, 443–450 (1968).

[b20] Friedman L. & Holstein T. Studies of polaron motion: Part III: the Hall mobility of the small polaron. Ann. Phys. 21, 494–549 (1963).

[b21] Holstein T. Studies of polaron motion: Part I. The molecular-crystal model. Ann. Phys. 8, 325–342 (1959).

[b22] Yamashita J. & Kurosawa T. On electronic current in NiO. J. Phys. Chem. Solids 5, 34–43 (1958).

[b23] Yamanaka S. . Thermophysical properties of perovskite-type strontium cerate zirconate. J. Am. Ceram. Soc. 88, 1496–1499 (2005).

[b24] Landau L. D. Über Die Bewegung der Elektronen in Kristallgitter. Phys. Z. Sowjet. 3, 644–645 (1933).

[b25] Pekar S. I. Teoriya polyaronov. Zh. Eksp. Teor. Fiz.+ 19, 796–806 (1949).

[b26] March N. H. & Parrinello M. Collective Effects in Solids and Liquids, (Graduate Student Series in Physics) Adam Hilger Ltd (1982).

[b27] Fischer S. F., Hofacker G. L. & Ratner M. A. Spectral behavior of hydrogen‐bonded systems: quasiparticle model. J. Chem. Phys. 52, 1934–1947 (1970).

[b28] Fischer S. F., Hofacker G. L. & Sabin J. R. Proton-phonon coupling in a hydrogen bonded system. Phys. Kondens. Mater. 8, 268–278 (1969).

[b29] Samgin A. L. Lattice-assisted proton hopping in oxides at low temperatures. J. Phys. Chem. Solids 74, 1661–1668 (2013).

[b30] Samgin A. L. Mechanism of high-temperature protonic conduction in the SrCeO_3_ oxides. Russ. J. Electrochem. 35, 287–293 (1999).

[b31] Samgin A. L. & Ezin A. N. Room-temperature proton-hopping transport in rutile-type oxides in the field of resonant laser radiation. Tech. Phys. Lett. 40, 252–255 (2014).

[b32] Kreuer K. D. in: Perovskite Oxide for Solid Oxide Fuel Cells 261–267Springer (2009).

[b33] Wakamura K. Ion conduction in proton- and related defect (super) ionic conductors: Mechanical, electronic and structure parameters. Solid State Ionics 180, 1343–1349 (2009).

[b34] Wakamura K. Roles of phonon amplitude and low-energy optical phonons on superionic conduction. Phys. Rev. B 56, 11593–11599 (1997).

[b35] Wakamura K. in: Physics of Solid State Ionics Research Signpost (2006).

[b36] Wakamura K. & Uemori H. Relationship between optical dielectric constant and ionic activation energy in perovskite-type proton conductors BaCe1-xMxO3 (M=Nd and Gd ). J. Phys. Soc. Jpn 79, 37–41 (2010).

[b37] Chen Q. . Effect of compressive strain on the raman modes of the dry and hydrated BaCe_0.8_Y_0.2_O_3_ proton conductor. J. Phys. Chem. C 115, 24021–24027 (2011).

[b38] Samgin A. L. Optically stimulated proton hopping in oxides at low temperatures. Solid State Commun. 152, 585–587 (2012).

[b39] Firsov Y. A. Small Polarons in: Polarons in Advanced Materials Springer (2007).

[b40] Spahr E. J. . Giant enhancement of hydrogen transport in rutile TiO_2_ at low temperatures. Phys. Rev. Lett. 104, 205901 (2010). 20867041 10.1103/PhysRevLett.104.205901

[b41] Hempelmann R. . Quasi-elastic neutron-scattering study of proton diffusion in SrCe_0.95_Yb_0.05_H_0.02_O_2.985_. Solid State Ionics 77, 152–156 (1995).

[b42] Malavasi L., Ritter C. & Chiodelli G. Correlation between thermal properties, electrical conductivity, and crystal structure in the BaCe_0.80_Y_0.20_O_2.9_ proton conductor. Chem. Mater. 20, 2343–2351 (2008).

[b43] Matzke T. . Quasielastic thermal neutron scattering experiment on the proton conductor SrCe_0.95_Yb_0.05_H_0.02_O_2.985_. Solid State Ionics 86-8, 621–628 (1996).

[b44] Savaniu C. D., Canales-Vazquez J. & Irvine J. T. S. Investigation of proton conducting BaZr_0.9_Y_0.1_O_2.95_: BaCe_0.9_Y_0.1_O_2.95_ core–shell structures. J. Mater. Chem. 15, 598–604 (2005).

[b45] Karlsson M. . Quasielastic neutron scattering of hydrated BaZr_0.90_A_0.10_O_2.95_ (A=Y and Sc). Solid State Ionics 180, 22–28 (2009).

[b46] Genet F., Loridant S. & Lucazeau G. Vibrational normal modes of the D-2h(16) phase of BaCeO_3_: a critical comparison of force fields. J. Raman Spectrosc. 28, 255–276 (1997).

[b47] Hermet J., Torrent M., Bottin F., Dezanneau G. & Geneste G. Oxide ion and proton transport in Gd-doped barium cerate: a combined first-principles and kinetic Monte Carlo study. J. Mater. Chem. A 2, 9055–9066 (2014).

[b48] Sewell G. L. Model of thermally activated hopping motion in solids. Phys. Rev 129, 597–608 (1963).

[b49] Larsen D. M. Intermediate-coupling polaron effective mass. Phys. Rev. 174, 1046–1049 (1968).

[b50] Mesot J., Janssen S., Holitzner L. & Hempelmann R. FOCUS: time-of-flight spectrometer for cold neutron at SINQ. J. Neutron Res. 3, 293–310 (1996).

[b51] Janssen S., Mesot J., Holitzner L., Furrer A. & Hempelmann R. FOCUS: a hybrid TOF-spectrometer at SINQ. Phys. B 234-236, 1174–1176 (1997).

[b52] Azuah R. T. . DAVE: a comprehensive software suite for the reduction, visualization, and analysis of low energy neutron spectroscopic data. J. Res. Natl Inst. Stan. Technol. 114, 341 (2009). 10.6028/jres.114.025 PMC4646530 27504233

[b53] Bagdassarov N., Freiheit H. C. & Putnis A. Ionic conductivity and pressure dependence of trigonal-to-cubic phase transition in lithium sodium sulphate. Solid State Ionics 143, 285–296 (2001).

