# Supplementary material for: Experimental neutron scattering evidence for proton polaron in hydrated metal oxide proton conductors
Source: Nat Commun. 2017 Jun 14;8:15830. doi: 10.1038/ncomms15830 (PMC5474746; doi:10.1038/ncomms15830)
Supplement: Supplementary Information [file ncomms15830-s1.pdf]

Type of file: PDF
Size of file: 0 KB
Title of file for HTML: Supplementary Information
Description: Supplementary Figures and Supplementary References

Supplementary Information

Supplementary Figures

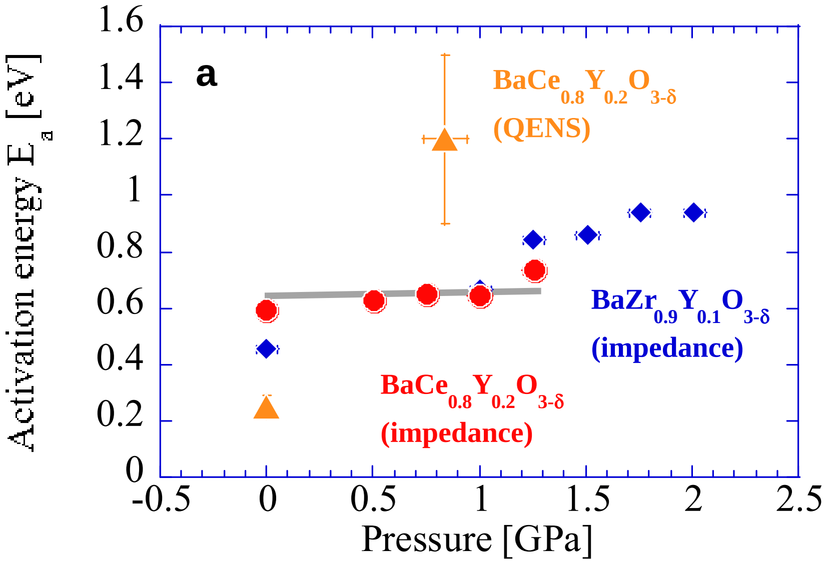

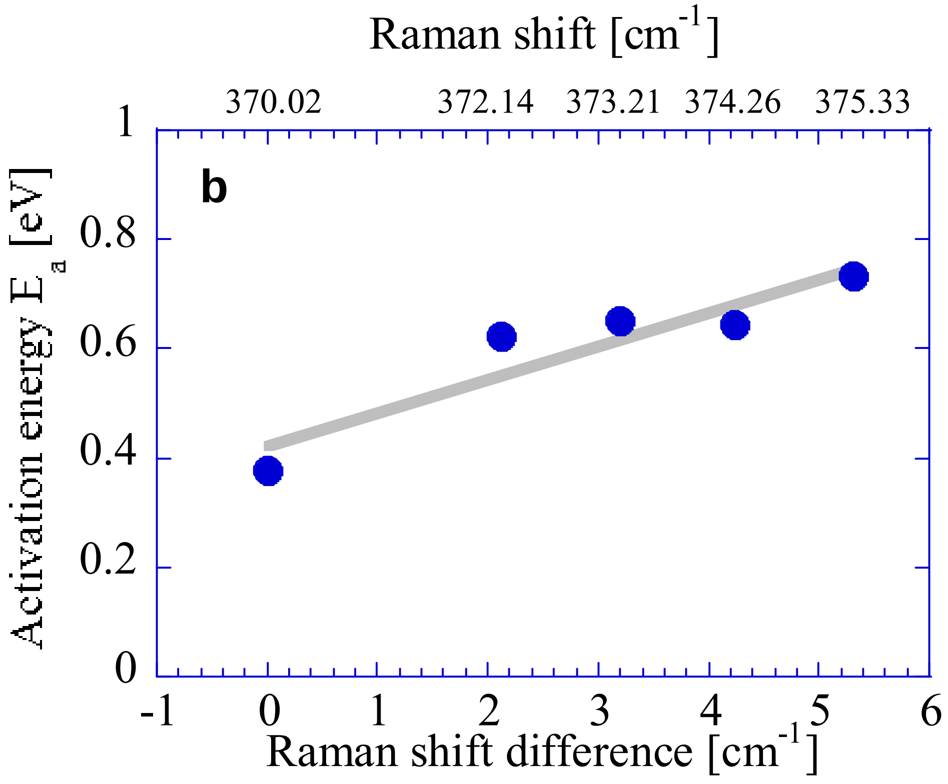

Supplementary Figure 1 | Activation energy vs pressure and Raman shift. (**a**) Activation energy determined from high pressure EIS (filled red circle), and from QENS (filled orange triangle, two data points) for hydrated BCY20 and hydrated $BaZr_{0.9}Y_{0.1}O_{3-\delta}$ (filled blue diamond) as a function of pressure. Solid gray line is the calculation from equation (5) using a coupling constant u = 5.29 (Table 1, line 1), ω taken from Ce-O stretching mode obtained from high pressure Raman data in [1]. (**b**) Variation of the activation energy with Raman shift under high pressure as obtained from hydrated BCY20 with impedance [2] and Raman spectroscopy [1].

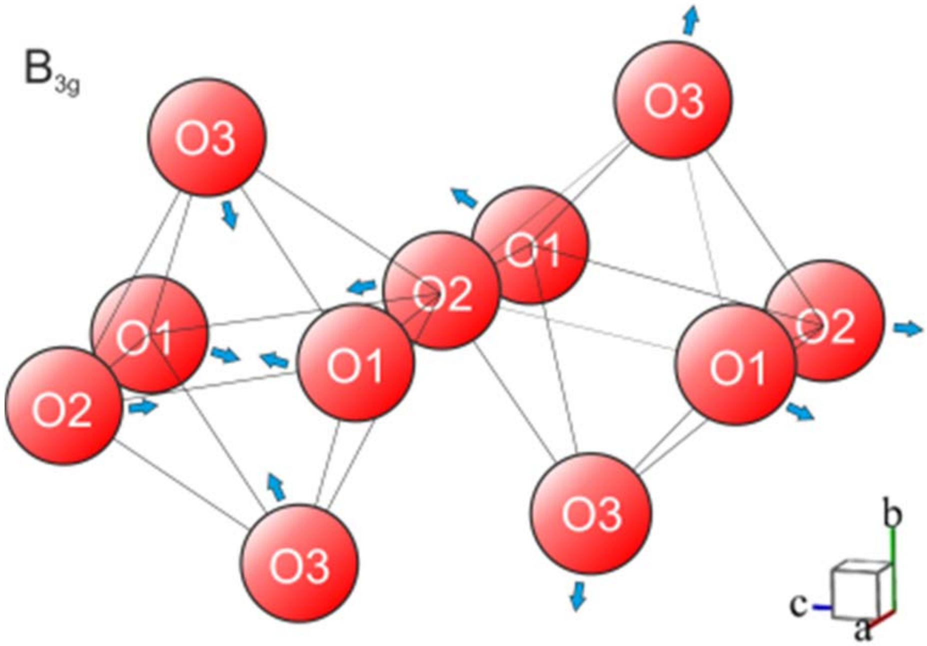

Supplementary Figure 2 | Description of the breathing mode. Schematic of Ce-O B_{3g} stretching mode, corresponding to the “breathing” motion of octahedron.

Supplementary references

1. Chen Q., *et al.* Effect of Compressive Strain on the Raman Modes of the Dry and Hydrated BaCe0.8Y0.2O3 Proton Conductor. *J. Phys. Chem. C.* **115**, 24021-24027 (2011).

2. Chen Q., Braun A., Yoon S., Bagdassarov N. & Graule T. Effect of lattice volume and compressive strain on the conductivity of BaCeY-oxide ceramic proton conductors. *J. Eur. Ceram. Soc.* **31**, 2657-2661 (2011).
